# Nonanal Stimulates Growth Factors via Cyclic Adenosine Monophosphate (cAMP) Signaling in Human Hair Follicle Dermal Papilla Cells

**DOI:** 10.3390/ijms21218054

**Published:** 2020-10-28

**Authors:** Soyoon Park, Wesuk Kang, Dabin Choi, Bomin Son, Taesun Park

**Affiliations:** Department of Food and Nutrition, Brain Korea 21 PLUS Project, Yonsei University, 50 Yonsei-ro, Seodaemun-gu, Seoul 120-749, Korea; thdbs1201@naver.com (S.P.); wesuk42@naver.com (W.K.); vin1411@naver.com (D.C.); mim1110@naver.com (B.S.)

**Keywords:** nonanal, cAMP, growth factor, dermal papilla cell

## Abstract

Human hair follicle dermal papilla cells (DPCs) are a specialized population of cells located in the hair follicles and regulate hair growth and development, particularly by releasing numerous growth factors in response to various physiological conditions. In the present study, we aimed to test whether nonanal, a scent compound from plants, stimulated growth factors in DPCs and to delineate the underlying mechanisms involved. We found that nonanal promoted DPC proliferation in a dose-dependent manner. Meanwhile, it also increased the intracellular cyclic adenosine monophosphate (cAMP) levels and the expression of various growth factor genes such as vascular endothelial growth factor, keratinocyte growth factor, and insulin-like growth factor 1. Furthermore, nonanal treatment stimulated DPC migration. Notably, the benefits of nonanal use were abrogated by cAMP inhibition. Our results reveal the potential of nonanal in preventing hair loss and suggest that its effects are cAMP-mediated in DPCs.

## 1. Introduction

Hair is an epidermal outgrowth composed of keratins originating from hair follicles deep down in the dermis. These hair follicles have been shown to produce increased amounts of keratin in response to growth factors; thus, growth factors are considered as key molecules that participate in hair growth initiation and suppression [1,2,3]. Dermal papilla, located at the base of the hair follicle, has its own blood supply and thus plays a crucial role in regulating numerous growth factors in response to various physiological conditions [4,5]. It has been reported that various causes of hair loss, including emotional stress, hormone imbalance, and nutritional deficiency, are characterized by the condensation of the dermal papilla along with impaired ability to produce growth factors [6,7,8].

At the cellular level, the production of growth factors is tightly regulated by several intracellular signaling pathways (e.g., Wnt/β-catenin and cyclic adenosine monophosphate (cAMP) that transmit a variety of extracellular stimuli. There are a few exogenous Wnt ligands that activate downstream β-catenin signaling, which leads to enhanced lymphoid enhancer-binding factor 1 (LEF1) transcription factor and contributes to growth factor gene enrichment [9,10,11]. Contrarily, cAMP, which can be activated by various agents targeting upstream G protein coupled receptor (GPCR; targeted by approximately 40% of all drugs), has been recognized as an important regulator of various growth factors, although the exact mechanism of growth factor regulation by cAMP has not been elucidated with consensus [12,13,14,15].

Cell proliferation might be a surrogate marker for increased growth factors in vitro [16,17,18], thus we attempted to explore the molecules that could enhance the proliferation of human hair follicle dermal papilla cells (DPCs). Since the beneficial effects of phytochemicals on hair growth have gained major interest, an in-house phytochemical library with about 500 entries, most of which have commercial usage such as food additives and cosmetic ingredients, was built as the first step. Then, through the screening process, we identified that nonanal induced a significant increase in the cell proliferation. Nonanal is a scent compound categorized as an aldehyde with the chemical formula C_9_H_18_O and has been widely reported in numerous natural essential oils, including cinnamon, rose, citrus, and pine [19,20]; however, its biological function remains unknown. This study aimed to investigate whether nonanal stimulated growth factors in DPCs and to delineate the mechanisms involved.

## 2. Results

### 2.1. Nonanal Increases Cell Proliferation

**Figure 1:**
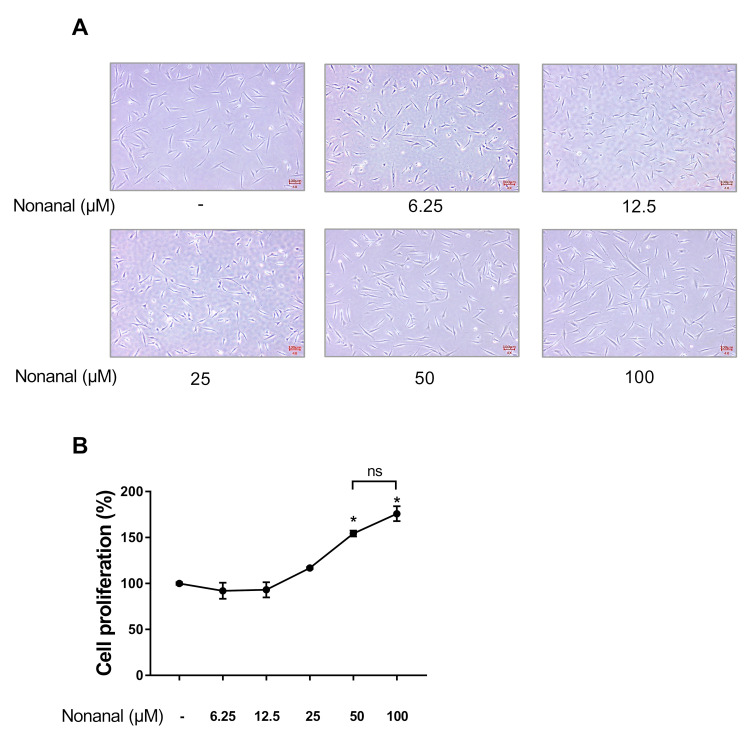
Effect of nonanal on dermal papilla cell (DPC) proliferation. DPCs were treated with vehicle (dimethyl sulfoxide; DMSO) or indicated concentrations of nonanal ranging from 6.25 to 100 µM. (**A**) Representative images of cells in response to nonanal treatment at indicated concentrations for 24 h were captured. (**B**) Cell proliferation was determined in response to nonanal treatment at indicated concentrations for 24 h by WST-1 assay. (**C**) DPCs were exposed to 50 µM of nonanal at different time points (12, 24, and 48 h) and cell proliferation was determined by WST-1 assay. Data were statistically analyzed using the Student’s *t*-test. Data are presented as mean ± standard error of mean (SEM) of the results of three independent experiments. * *p* < 0.05 vs. control.

To evaluate whether nonanal affected cell proliferation in DPCs, we treated DPCs with either vehicle (dimethyl sulfoxide; DMSO) or various concentrations of nonanal ranging from 6.25 to 100 µM. After 24 h, the cells were observed using a microscope and a water-soluble tetrazolium salt (WST-1) assay was conducted (Figure 1A,B). Treatment of DPCs with nonanal significantly enhanced cell proliferation in a dose-dependent manner compared to that in vehicle controls. The proliferative effect seemed to be saturated at 50 µM nonanal in 24 h time point (Figure 1B,C); therefore, all further experiments were performed with 50 µM nonanal. On the other hand, nonanal treatment did not affect the proliferation of Hs68 dermal fibroblasts at 50 µM concentration (Figure S1). 

### 2.2. Nonanal Enhances Gene Expression of Growth Factors

**Figure 2:**
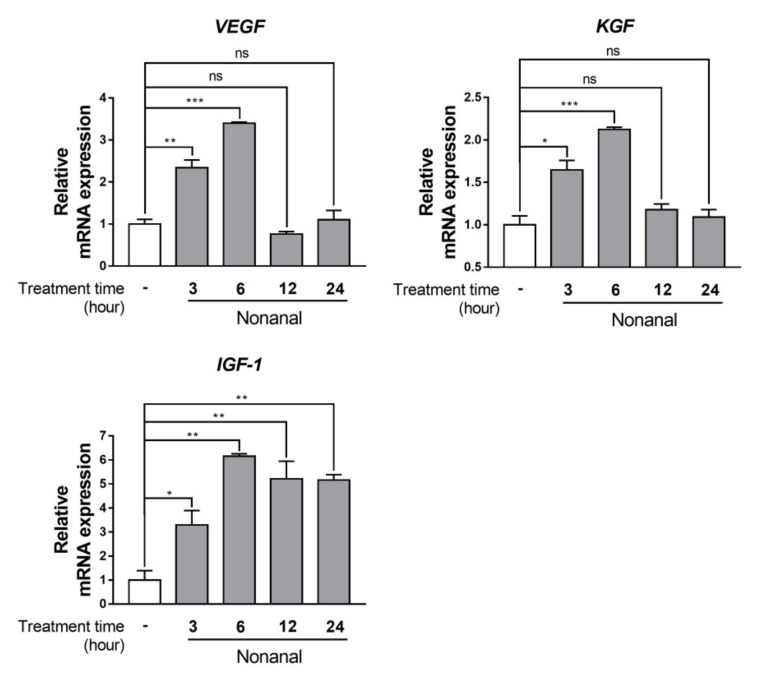
The time-course of mRNA expression of growth factors in response to nonanal treatment in DPCs. DPCs were treated with vehicle or 50 µM nonanal and the relative mRNA levels of growth factors were measured by real-time PCR over a 24 h time-course. Data were statistically analyzed using the Student’s *t*-test. Data are presented as mean ± SEM of the results of three independent experiments. * *p* < 0.05, ** *p* < 0.01, *** *p* < 0.001. ns, not significant.

DPCs were treated with either vehicle or nonanal for 3, 6, 12, and 24 h. The mRNA expression levels of growth factors including vascular endothelial growth factor (*VEGF*), keratinocyte growth factor (*KGF*), and insulin-like growth factor 1 (*IGF-1*) in DPCs were then analyzed (Figure 2). Nonanal treatment significantly increased mRNA expressions of all growth factors studied, which peaked at 6 h, compared with vehicle controls.

### 2.3. Nonanal Does Not Affect the β-Catenin Signaling Pathway

**Figure 3:**
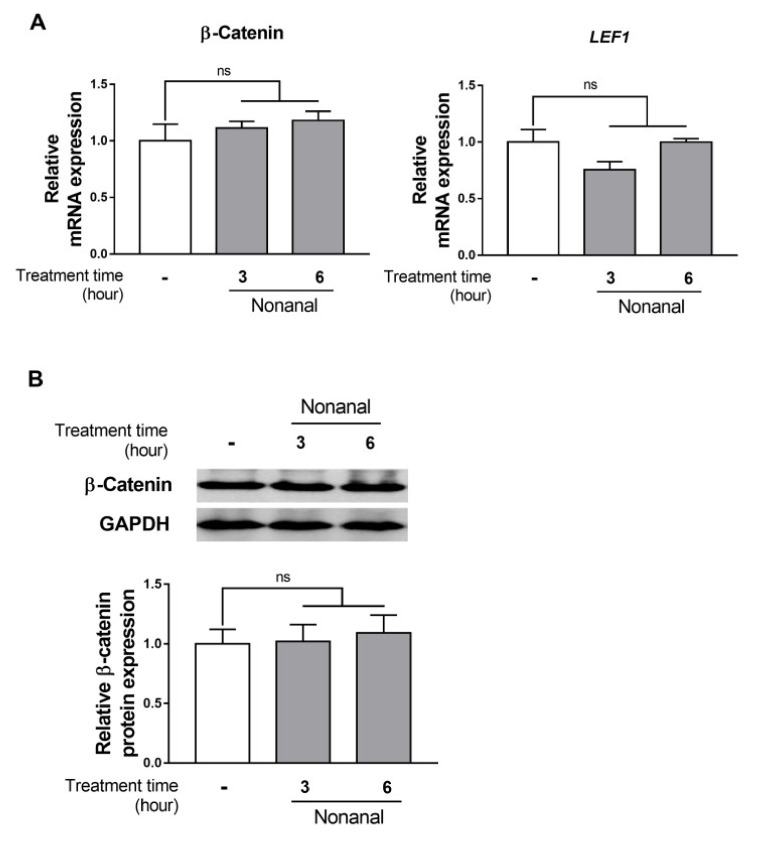
Effect of nonanal on β -catenin pathway in DPCs. DPCs were exposed to vehicle or 50 µM of nonanal at different time points (3 and 6 h). (**A**) Relative β-catenin and lymphoid enhancer-binding factor 1 (*LEF1*) mRNA levels were analyzed by real-time PCR. (**B**) β-catenin protein levels were detected by Western blot. Data were statistically analyzed using the Student’s *t*-test. Data are presented as mean ± SEM of the results of three independent experiments. ns, not significant.

To explore the participation of β-catenin signaling in the nonanal-induced growth, β-catenin and its downstream effector, *LEF1*, expression levels were assessed in DPCs. DPCs were exposed to either vehicle or nonanal for 3 and 6 h. Nonanal treatment did not significantly affect β-catenin and *LEF*1 mRNA expression (Figure 3A). Consistent with the gene expression results, there was no change in β-catenin protein expression after treating DPCs with nonanal for 3 and 6 h (Figure 3B).

### 2.4. Nonanal Activates the cAMP/PKA Signaling Pathway

**Figure 4:**
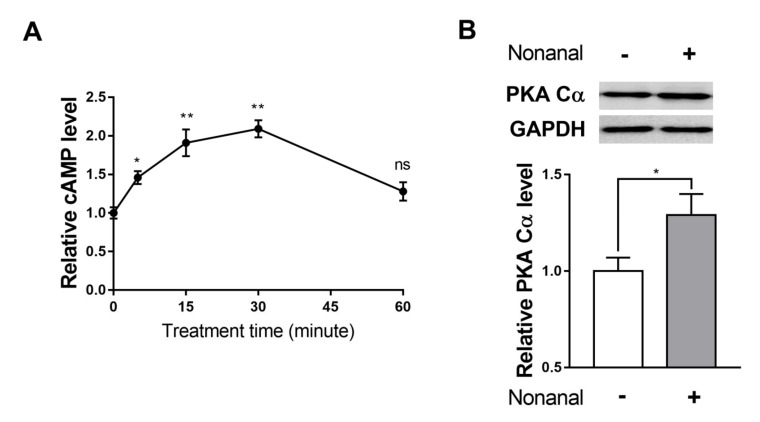
Effect of nonanal on the cyclic adenosine monophosphate (cAMP) pathway in DPCs. (**A**) DPCs were treated with vehicle or 50 µM nonanal for 5, 15, 30, and 60 min. Intracellular cAMP levels were determined using enzyme-linked immunosorbent assay (ELISA) kit. (**B**) The protein levels of protein kinase A catalytic subunit (PKA Cα) were detected by Western blotting after treatment with vehicle or 50 µM nonanal for 30 min. Data were statistically analyzed using the Student’s *t*-test. Data are presented as mean ± SEM of the results of three independent experiments. * *p* < 0.05, ** *p* < 0.01. ns, not significant.

To investigate the role of the cAMP/ protein kinase A (PKA) pathway in the nonanal-induced growth, intracellular cAMP level and protein expression of PKA catalytic subunit α (PKA Cα), which is activated by cAMP, were evaluated in DPCs. DPCs were treated with nonanal for 5, 15, 30, and 60 min. The cAMP level significantly elevated at 5, 15, and 30 min and was maximum at 30 min, which then declined (Figure 4A). Furthermore, nonanal treatment significantly enhanced PKA Cα protein expression in DPCs compared with vehicle controls (Figure 4B).

### 2.5. cAMP Is Involved in Nonanal-Induced DPC Proliferation

**Figure 5:**
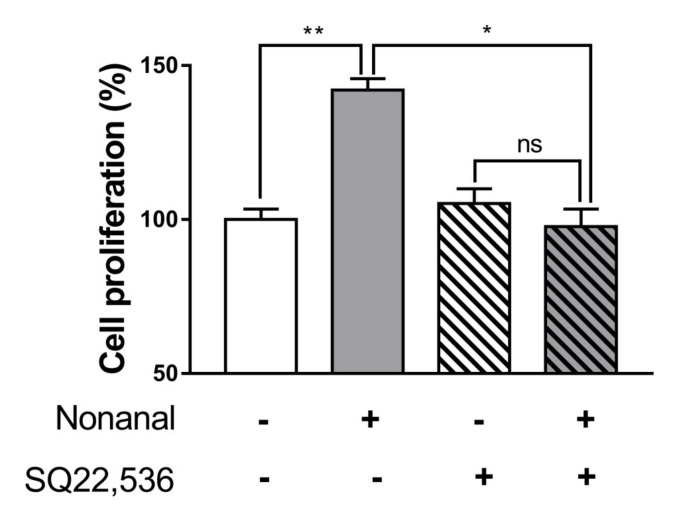
Effect of inhibiting cAMP production on nonanal-stimulated proliferation of DPCs. DPCs were treated with vehicle or 50 µM nonanal for 24 h. The vehicle or 50 µM SQ22,536, a cAMP inhibitor, was added 1 h before nonanal treatment. Cell proliferation was determined by WST-1 assay. Data were statistically analyzed using the Student’s *t*-test. Data are presented as mean ± SEM of the results of three independent experiments. * *p* < 0.05, ** *p*< 0.01. ns, not significant.

Since nonanal increases the intracellular cAMP levels, the role of cAMP in nonanal-induced DPC proliferation was evaluated. Nonanal treatment significantly increased cell proliferation, which was almost completely blocked by treating the cells with cAMP inhibitor SQ22,536 (50 µM; Figure 5).

### 2.6. cAMP Is Implicated in Nonanal-Induced Gene Expression of Growth Factors

**Figure 6:**
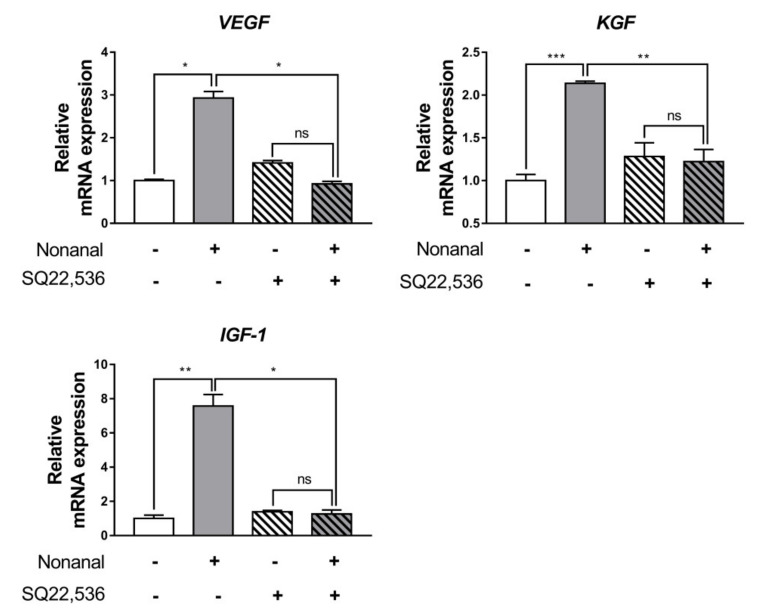
Effect of cAMP inhibitors on nonanal-stimulated mRNA expression of growth factors. DPCs were treated with vehicle or 50 µM nonanal for 6 h. The vehicle or 50 µM SQ22,536 was added 1 h before nonanal treatment. Relative mRNA levels of the growth factors were measured by real-time PCR. Data were statistically analyzed using the Student’s *t*-test. Data are presented as mean ± SEM of the results of three independent experiments. * *p* < 0.05, ** *p* < 0.01, *** *p* < 0.001. ns, not significant.

To investigate the role of cAMP in nonanal-induced gene expression of growth factors, we used 50 µM SQ22,536 to inhibit cAMP production in DPCs. The mRNA expression of VEGF, KGF, and IGF-1 was significantly enhanced by nonanal, but these effects are fully abolished by SQ22,536 (Figure 6).

### 2.7. Nonanal Promotes Cell Migration via cAMP

**Figure 7:**
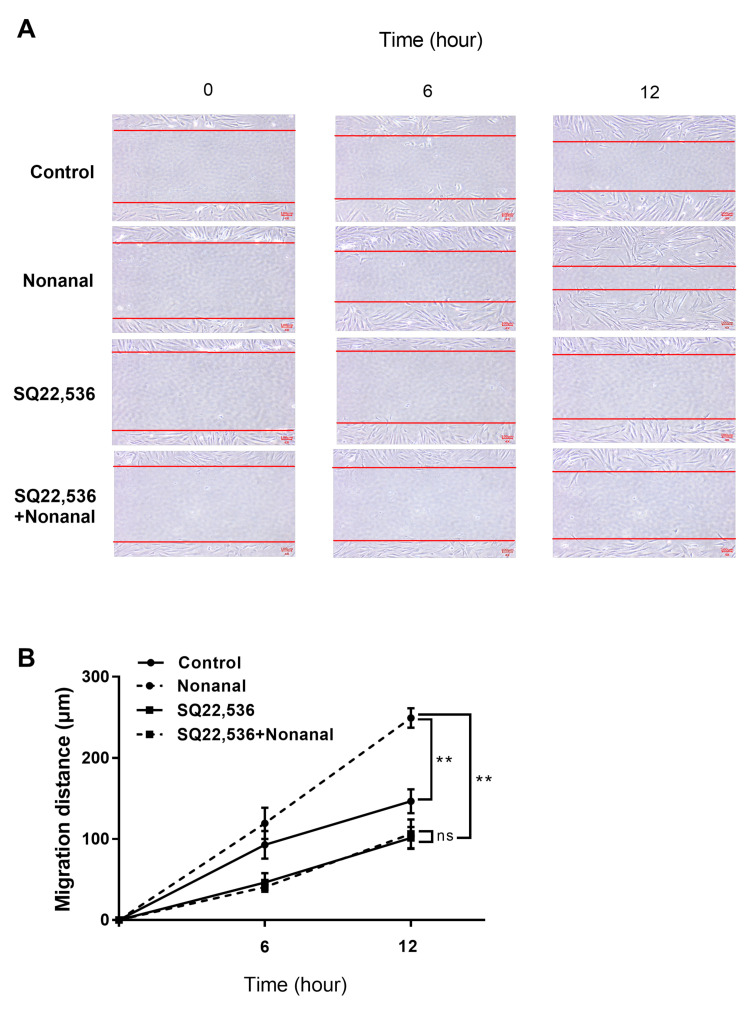
Effect of nonanal on DPC migration via the cAMP signaling pathway. Migration assay was conducted in confluent DPCs by drawing a straight line across the bottom of the dish, followed by incubation with either vehicle or 50 µM nonanal at different time points (0, 6, and 12 h). The vehicle or 50 µM SQ22,536 was added 1 h before nonanal treatment. (**A**) Phase-contrast microscopy images of the scratched area were captured. The lines delineate the migrating edges of cells. (**B**) Quantification of cell migration was performed by measuring the area occupied by the cells at different time points. Data were statistically analyzed using the Student’s *t*-test. Data are presented as mean ± standard error of mean (SEM) of the results of three independent experiments. ** *p* < 0.01. ns, not significant.

**Figure 8:**
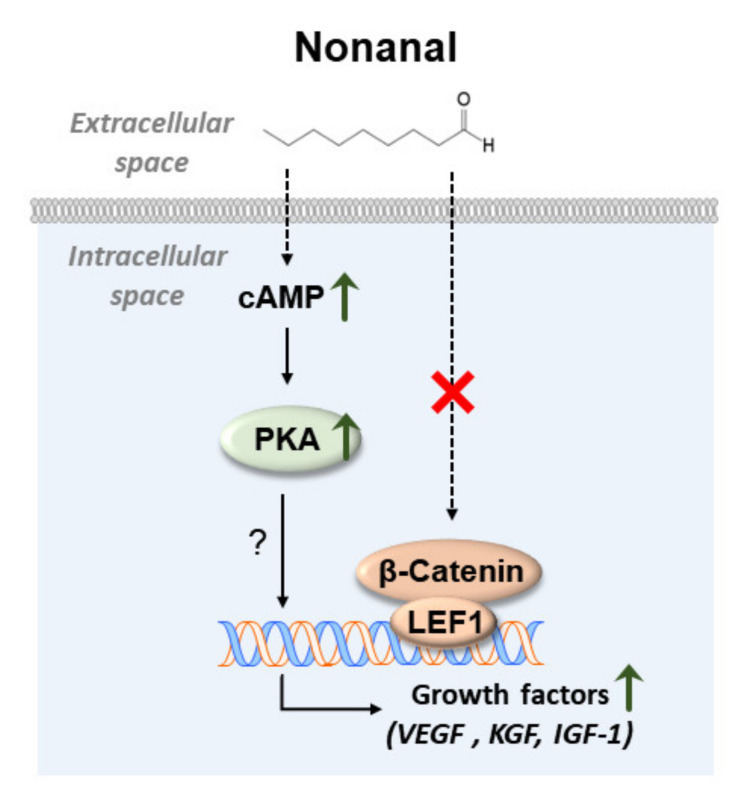
A suggested mechanism by which nonanal induces the growth factors expression in DPCs via the cAMP signaling pathway. As shown in a schematic illustration, stimulation of the cAMP signaling pathway in response to nonanal increases levels of growth factors such as VEGF, KGF, and IGF-1. The detailed molecular mechanism by which cAMP-mediated growth factor secretion increases needs further investigation. Interestingly, β -catenin and LEF1 levels did not increase in response to nonanal treatment.

To determine whether nonanal affected DPC migration via cAMP, DPCs were treated with nonanal in the presence or absence of SQ22,536. Cell migration assay revealed that nonanal significantly increased migration in comparison with vehicle controls. However, nonanal-induced DPC migration was significantly inhibited by SQ22,536 (Figure 7A,B). Taken together, nonanal-induced cAMP pathway appears to contribute to the stimulation of growth factors in DPCs (Figure 8).

## 3. Discussion

Topical delivery of agents offers several advantages over oral delivery for treatment of extensive alopecia. There are fewer digestive enzymes, the pH is not extreme, and it shows considerable efficiency and bioavailability because of the proximity to the hair follicle. This may also lead to decreased systematic toxicity [21,22,23]. However, the skin epidermis can prevent the diffusion of many exogenous agents. It is commonly believed that only relatively low-molecular-weight (below 500 Da) lipophilic molecules can effectively permeate through the human skin [24]; nonanal is expected to be easily absorbed and reach the dermal papilla with topical application because of its lipophilicity and low molecular weight (142.2 Da). In addition, this compound has already been used in the cosmetic industry as a fragrance ingredient with no reports of skin irritation or sensitization [25,26,27].

It has been accepted that DPC has an essential role in the regulation of hair growth owing to its ability to secrete various growth factors. For example, KGF acts exclusively on the epidermis, where it stimulates the synthesis of hair keratin by inducing keratinocyte proliferation, migration, and differentiation [28,29]. However, several other growth factors act not only on the epidermis, but also on other locations in the skin for hair growth promotion. VEGF, as its name suggests, additionally increases vascularization around the hair follicles, thereby providing adequate nutrition and oxygen to the dermal papilla [30,31,32]. IGF-1, besides the epidermis and blood vessels, can act on the producer cell itself (autocrine signaling), which is known to strongly enhance DPC proliferation and migration [33,34,35]. In the present study, nonanal promotes proliferation and migration of DPCs. While the upregulation of growth factors such as IGF-1, KGF, and VEGF is a compelling potential mechanism, cAMP could also result in proliferation and migration through a number of mechanisms. Further study is needed to determine whether the increased levels of these factors are actually responsible for proliferation and migration induced by nonanal compound. If nonanal functions in the human scalp tissues in a way which is similar to that in DPCs, then the phenotypic improvement of hair loss may be accelerated through the simultaneous release of these growth factors.

In the present study, nonanal treatment stimulated DPC migration, which is known to be positively regulated by growth factors [18,36,37]. Cell migration, characterized by the orchestrated movements of cells in particular directions, is crucial in many developmental and morphogenic processes controlling organ size [38,39]. It has been shown that increased DPC migration can lead to the enhancement of dermal papilla volume, which determines hair follicle size and subsequently affects hair shaft thickness [40,41,42]. One clinical study has shown that the DPCs derived from bald scalps exhibit impaired migration activity [43].

We found that nonanal treatment did not alter β-catenin signaling, but activated the cAMP signaling pathway in DPCs (Figure 3 and Figure 4). Although mechanisms by which nonanal enhances the cAMP level are unknown, it is noteworthy that nonanal has been identified as a ligand of numerous olfactory receptors (ORs; e.g., OR1G1, OR52D1, and OR1A1) which constitute the largest GPCR subfamily, in each OR-transfected HEK293 and HeLa cells [44,45]. GPCR activation in the membrane can effectively stimulate adenylyl cyclase that catalyzes ATP to cAMP conversion [46,47]. The beneficial effects of nonanal seem to be specific to cell types as nonanal treatment did not affect the proliferation of Hs68 dermal fibroblasts, one of the major skin cell types. Further research is required to elucidate the exact mechanism by which nonanal activates the cAMP signaling in DPCs.

## 4. Materials and Methods

### 4.1. Cell Culture

Human follicle DPCs (55, female, Caucasian) were obtained from PromoCell (Heidelberg, Germany). These cells were isolated from human dermis of the scalp, originating from the occipital region. The cells were cultured in DPC growth medium with supplement mix (PromoCell) and 1% penicillin-streptomycin (10,000 U/mL; Gibco; Grand Island, NE, USA). The cells were incubated at 37 °C in 5% carbon dioxide humidified air and passaged every 3 days using 4-(2-hydroxyethyl)-1-piperazine ethanesulfonic acid (HEPES) buffer, trypsin/ethylene diamine tetra acetic acid (EDTA) solution, and trypsin neutralizing solution (PromoCell). Initial passage number of cells was 2, and the cells from passage numbers 3 to 5 were used for the study. Hs68 human dermal fibroblasts (American Type Culture Collection (ATCC), Manassas, VA, USA) were cultured in high-glucose Dulbecco’s modified Eagle’s medium (Hyclone, Logan, UT, USA) supplemented with 10% of fetal bovine serum (Hyclone) and 1% penicillin-streptomycin. Hs68 cells from passage numbers 5 to 7 were used for the study.

### 4.2. WST-1 Assay

WST-1 assay was performed to assess cell proliferation. DPCs were seeded in a 96-well plate at a density of 3000 cells per well and treated with vehicle (DMSO; Sigma-Aldrich; St. Louis, MO, USA) or various concentrations of nonanal (Sigma-Aldrich). When required, vehicle (DMSO) or SQ22,536 (Sigma-Aldrich), a cAMP inhibitor, was added 1 h before nonanal treatment. Images of the cells were captured using a Nikon Eclipse TS2 microscope equipped with the DMX1200 camera (Nikon, Tokyo, Japan). Subsequently, the treated cells were incubated for 2 h with the WST-1 reagent (Sigma-Aldrich) diluted 1:10 in culture medium. Absorbance was read at 440 nm using the Infinite M200 microplate reader (Tecan; Männedorf, Switzerland). In all the experiments, the final DMSO concentration was less than 0.1% (*v*/*v*). Furthermore, to determine whether the proliferation of Hs68 was also affected by nonanal treatment, the same method was employed as above.

### 4.3. cAMP Measurements

DPCs were seeded in a 12-well plate at a density of 40 000 cells per well and treated with nonanal at various time points. The cells were washed with HEPES buffer and lysed in 0.1 M HCl. Intracellular cAMP level in the lysates was measured using a cAMP enzyme-linked immunosorbent assay (ELISA) kit (Enzo Life Sciences; Farmingdale, NY, USA) following the manufacturer’s instructions. The cAMP level was normalized to the cellular protein content of the lysates, which was determined by the Bradford assay (BioRad; Hercules, CA, USA).

### 4.4. Real-Time PCR

DPCs were seeded in a 60 mm dish at a density of 200,000 cells and treated with vehicle or nonanal at various time points. When required, SQ22,536 was added 1 h before nonanal treatment. The RNA from the cells was isolated using the TRIzol reagent (Invitrogen; Carlsbad, CA, USA) and reverse-transcribed into cDNA with oligo(dT) primer (Invitrogen) and SuperScript IV reverse transcriptase (Invitrogen). The cDNA was amplified with gene-specific primers (Table 1) and SYBR green supermix (BioRad) on a CFX real-time system (BioRad). Data were analyzed using the comparative cycle threshold (Ct) method for relative quantitation and normalized to the levels of the internal control, glyceraldehyde 3-phosphate dehydrogenase (*GAPDH*).

### 4.5. Western Blotting

Western blotting was performed to determine the protein levels. DPCs were seeded in a 60-mm dish at a density of 200,000 cells, treated with either the vehicle or nonanal, and harvested at various time points. The cells were washed with HEPES buffer and lysed in a PRO-PREP protein extraction buffer (iNtRON; Seoul, Korea). Overall, 20 µg of protein was separated by sodium dodecyl sulfate-polyacrylamide gel electrophoresis (SDS-PAGE) and transferred onto a nitrocellulose membrane (Whatman; Kent, UK). Subsequently, the membrane was blocked with 5% bovine serum albumin (BSA; MP biomedicals; Irvine, CA, USA) in tris-buffered saline/Tween-20 (TBST) for 1 h and then incubated with a 1:1000-diluted anti-PKA Cα, anti-β-catenin, or anti-GAPDH antibodies (Cell Signaling; Herts, UK) overnight at 4 °C. The membrane was further incubated with a peroxidase-conjugated secondary antibody (Santa Cruz Biotechnology; Santa Cruz, CA, USA) for 1 h at 20 °C. Antibody binding was detected with electrochemiluminescence detection reagent (ECL; BioRad) and visualized using Ez-capture (ATTO; Tokyo, Japan). The protein levels were normalized to those of the loading control (GAPDH).

### 4.6. Migration Assay

DPCs were seeded in a 24-mm dish at a density of 20,000 cells per well and grown to confluence. On the day of the experiment, to inhibit cell proliferation, the cells were washed with HEPES buffer and preincubated with mitomycin C (15 μg/mL, Sigma-Aldrich) for 2 h. Subsequently, the cells were scratched in straight line with a sterile 200 μL tip. The cells were re-washed with HEPES buffer to remove cellular debris and incubated with vehicle or nonanal for 0, 6, and 12 h. When required, SQ22,536 was added 1 h before nonanal treatment. The same fields were photographed at each time point, and the area migrated by DPCs was measured using ImageJ (National Institutes of Health; NIH; Bethesda, MD, USA). 

### 4.7. Statistical Analysis

All data are presented as mean ± standard error of mean (SEM). Statistical analysis was performed by the Student’s *t*-test using SPSS Statistics 24 (SPSS; Chicago, IL, USA). Differences were considered as significant at *p* < 0.05.

## 5. Conclusions

In summary, we have demonstrated that nonanal increases the growth factor levels through cAMP-mediated signaling in DPCs. Although further in vivo experimental models should be tested to confirm the in vivo relevance of these in vitro findings, the current study highlights the potential utility of nonanal for treating hair loss associated with various physiological states and provides insights into the molecular mechanisms of the effects of nonanal in DPCs.

## Figures and Tables

**Table 1:** Primer sequences used for real-time PCR.

Gene Name	Primer Sequences (5′→ 3′)
Catenin beta 1 (*CTNNB1*)	F: CCGACACCAAGAAGCAGAGATG R: GTGGGATGGTGGGTGTAAGAG
Glyceraldehyde 3-phosphate dehydrogenase (*GAPDH*)	F: AAATCAAGTGGGGCGATGC R: AGGGGGCAGAGATGATGACC
Insulin-like growth factor-1 (*IGF-1*)	F: TTGCTCTCAACATCTCCCATCT R: TGCATCTTCACCTTCAAGAAAT
Keratinocyte growth factor (*KGF*)	F: AGAGAAGGGATGCTGGAGGT R: CGTAAGGGGCACTGTTTTAGAG
Lymphoid enhancer factor 1 (*LEF1*)	F: CAACCCTACCCATCCTCACTG R: GGCTCCTGCTCCTTTCTCTGT
Vascular endothelial growth factor (*VEGF*)	F: CTTCTGAGTTGCCCAGGAGA R: GGATGGAGGAAGGTCAACCA

## Supplementary Materials

Supplementary materials can be found at https://www.mdpi.com/1422-0067/21/21/8054/s1.
