# Supplementary material for: Nonanal Stimulates Growth Factors via Cyclic Adenosine Monophosphate (cAMP) Signaling in Human Hair Follicle Dermal Papilla Cells
Source: Int J Mol Sci. 2020 Oct 28;21(21):8054. doi: 10.3390/ijms21218054 (PMC7662673; doi:10.3390/ijms21218054)
Supplement: Supplementary file 1 [file ijms-21-08054-s001.zip › Supplementary_Material (201021).docx]

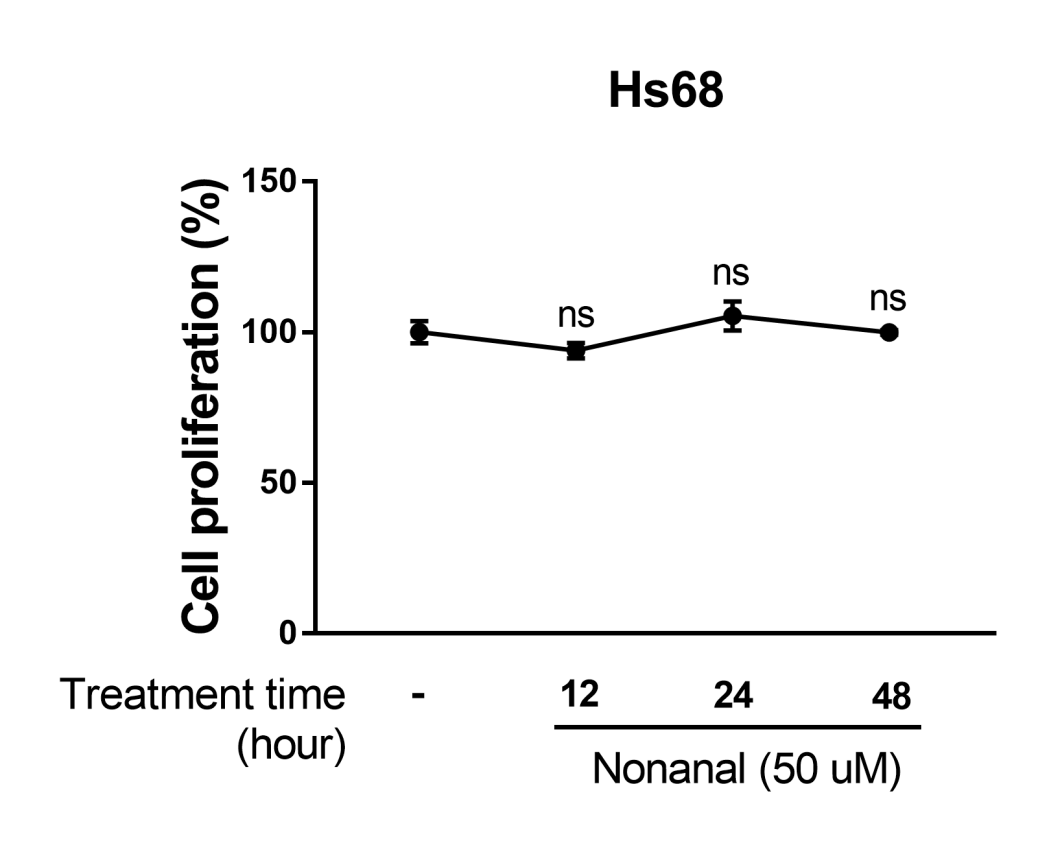


Figure S1. Nonanal treatment did not affect the proliferation of Hs68 dermal fibroblasts. The cells were treated with nonanal (50 µM) at different time points (12, 24, and 48 h) and cell proliferation was determined by WST-1 assay. The data was statistically analyzed using the Student’s t-test. Data are presented as the mean ± SEM of three separate experiments. ns, not significant.
