# Supplementary figures and images for: Nonanal Stimulates Growth Factors via Cyclic Adenosine Monophosphate (cAMP) Signaling in Human Hair Follicle Dermal Papilla Cells
Source: Int J Mol Sci. 2020 Oct 28;21(21):8054. doi: 10.3390/ijms21218054 (PMC7662673; doi:10.3390/ijms21218054)

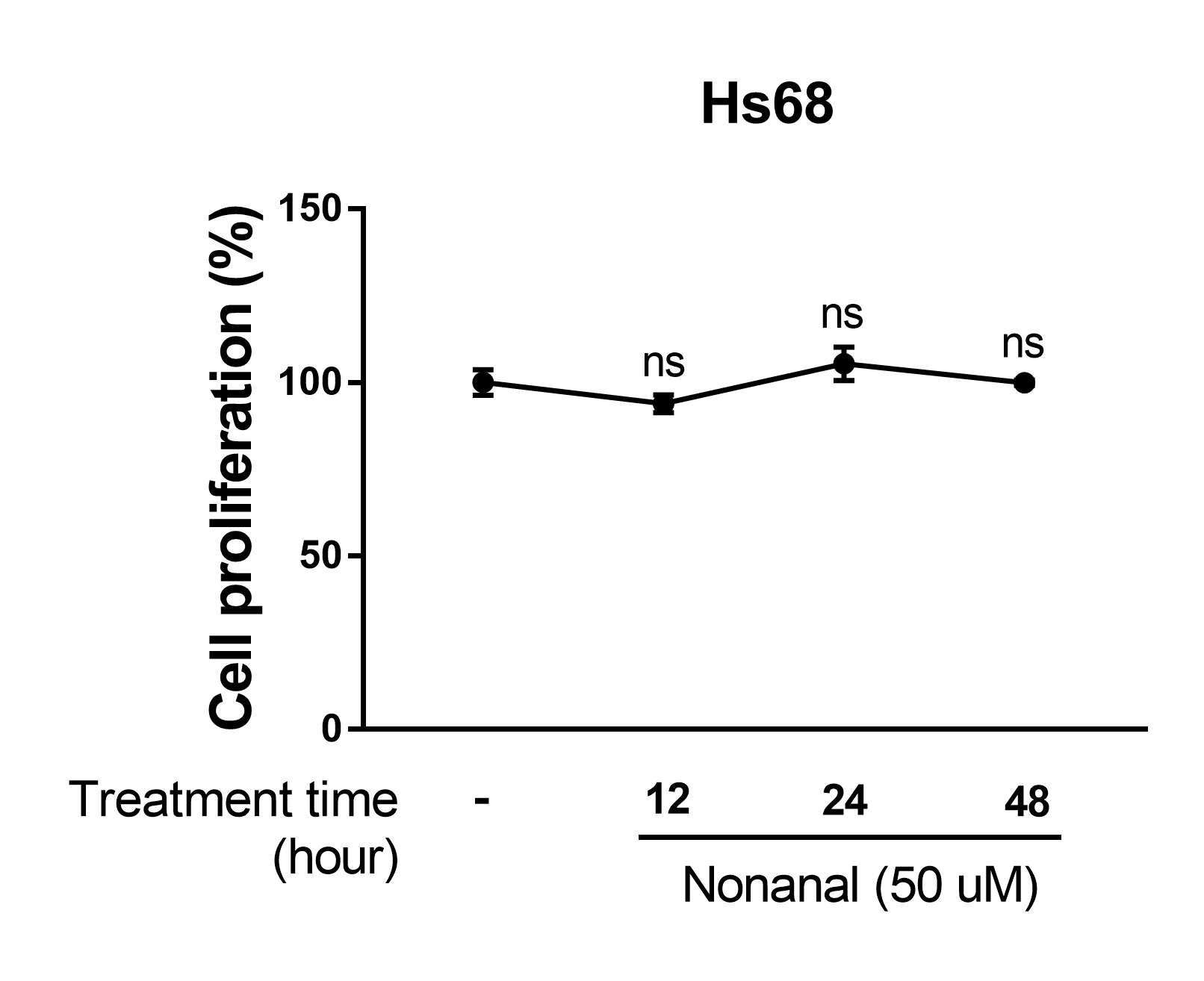

Supplement: Supplementary file 1 [file ijms-21-08054-s001.zip › Supplementary figure 1.tif]
